# A metabolic reprogramming-related prognostic risk model for clear cell renal cell carcinoma: From construction to preliminary application

**DOI:** 10.3389/fonc.2022.982426

**Published:** 2022-09-13

**Authors:** Qian Zhang, Lei Ding, Tianren Zhou, Qidi Zhai, Chenbo Ni, Chao Liang, Jie Li

**Affiliations:** Department of Urology, The First Affiliated Hospital of Nanjing Medical University, Nanjing, China

**Keywords:** clear cell renal cell carcinoma, metabolic reprogramming, weighted correlation network analysis, prognostic model, key gene

## Abstract

Metabolic reprogramming is one of the characteristics of clear cell renal cell carcinoma (ccRCC). Although some treatments associated with the metabolic reprogramming for ccRCC have been identified, remain still lacking. In this study, we identified the differentially expressed genes (DEGs) associated with clinical traits with a total of 965 samples *via* DEG analysis and weighted correlation network analysis (WGCNA), screened the prognostic metabolism-related genes, and constructed the risk score prognostic models. We took the intersection of DEGs with significant difference coexpression modules and received two groups of intersection genes that were connected with metabolism *via* functional enrichment analysis. Then we respectively screened prognostic metabolic-related genes from the genes of the two intersection groups and constructed the risk score prognostic models. Compared with the predicted effect of clinical grade and stage for ccRCC patients, finally, we selected the model constructed with genes of ABAT, ALDH6A1, CHDH, EPHX2, ETNK2, and FBP1. The risk scores of the prognostic model were significantly related to overall survival (OS) and could serve as an independent prognostic factor. The Kaplan-Meier analysis and ROC curves revealed that the model efficiently predicts prognosis in the TCGA-KIRC cohort and the validation cohort. Then we investigated the potential underlying mechanism and sensitive drugs between high- and low-risk groups. The six key genes were significantly linked with worse OS and were downregulated in ccRCC, we confirmed the results in clinical samples. These results demonstrated the efficacy and robustness of the risk score prognostic model, based on the characteristics of metabolic reprogramming in ccRCC, and the key genes used in constructing the model also could develop into targets of molecular therapy for ccRCC.

## Introduction

Renal cell carcinoma (RCC) is universal cancer, which accounts for 2.2% of the total cancer incidence and 1.8% of the total cancer mortality (1). According to The Cancer Statistics (2), at the end of 2021, about 76,080 new cases of RCC would be diagnosed, and 13780 patients with RCC would die in the USA. More than 40% of RCC patients will have metastasis after surgical intervention (3). Clear cell renal cell carcinoma (ccRCC), the prevailing and invasive histological subtype of RCC, has become a worldwide issue. Clinicians mainly employed the T, N, and M classification system and Fuhrman nuclear grade to prognosticate patients’ prognosis of ccRCC and guide clinical treatment decision-making. However, these prognostic tools require improvements and novel, robust, and specific prognostic models to acquire more accurate predictions.

A complex biological system cannot be changed by a single part of its components but by the interaction of these components. Bioinformatics, which introduces computational methods and mathematical models, enlarges the magnitude of data accumulated in the genomic, transcriptomic, and proteomic studies, allowing us to simulate the complexity of the biological system and understand these systems (4). Bioinformatics technologies have become increasingly prevalent in finding molecular mechanisms and specific biomarkers of diseases.

Weighted correlation network analysis (WGCNA) and differently expressed gene (DEG) analysis are increasingly being used as the analytical methods of bioinformatics. WGCNA is a system biology method for discovering modules of highly correlated genes and summarizing these modules by using the intramodular hub gene (5). Then, selecting important modules associated with clinical traits for further analysis. DEG analysis can find quantitative changes in gene expression levels and study molecular mechanisms of gene regulation. Using the combination of DEG and WGCNA could improve the accuracy of discriminating highly related candidate biomarker genes. Analyzing the genes that have been screened out, we found that these genes were primarily related to metabolism. Previous studies have shown a strong link between RCC and changes in metabolic pathways (6–8), and abnormally accumulated lipid droplets have been found in the ccRCC cytoplasm (9). Nevertheless, the prognostic effect of these metabolic genes on patients remains unclear.

In this study, we used WGCNA and DEG analyses to analyze the mRNA expression data of ccRCC from The Cancer Genome Atlas (TCGA) and Gene Expression Omnibus (GEO) databases, which showed differential co-expression genes, and to explore the relationship between these metabolic genes and prognosis of patients with ccRCC. Screening by WGCNA, we obtained the clinical traits of corelated genes, which may be considered as biomarkers and targets for treatment. Using prognostic metabolism-related genes, we constructed a prognostic prediction model and validated it.

## Materials and methods

The workflow of this study is shown in **Supplementary Figure S1**, and We will elaborate on each step in the following sub-sections.

### Acquisition datasets from TCGA and GEO databases

The gene expression dataset of ccRCC was downloaded from TCGA (https://portal.gdc.cancer.gov/) and GEO (https://www.ncbi.nlm.nih.gov/gds) databases. Before analysis of the dataset, patients with missing data of pathological diagnosis and corresponding clinical information were excluded. Afterward, the ccRCC dataset downloaded from the TCGA database included 611 samples and corresponding clinical information. The TCGA-KIRC dataset annotated using the Human hg38 gene standard track contains 72 normal counts, 539 tumor counts, and 19600 genes of RNAseq data.

Four datasets, including GSE36895, GSE46699, GSE53757, and GSE66270, were downloaded from the GEO database. The platform of such datasets is GPL570 [HG-U133_Plus_2] Affymetrix Human Genome U133 Plus 2.0 Array, which was used in gene probe annotation. We combined these four datasets into a single dataset and then normalized and cleaned the merged dataset using the R package affy (version 1.66.0), impute (version 1.62.0), limma (version 3.44.3), and sva (version 3.36.0) (10–12) (https://bioconductor.org/bioclite.R). The merged GEO-ccRCC dataset included 172 normal samples and 182 tumor samples. If one gene corresponded to duplicated probes, then we used the mean value of these probes.

### Identification of robust DEGs

TCGA-KIRC and GEO-ccRCC datasets were utilized for analysis. The ccRCC samples of patients were divided into two sets, normal and tumor samples. The R package limma (version 3.44.3) was used in analyzing the data and screening the DEGs with |logFC|>1 and ad. just P<0.05. By using R software, the DEGs of TCGA-KIRC and GEO-ccRCC datasets were visualized as a volcano plot, the abscissa and ordinate of which were adj.P and logFC, respectively. The upregulated genes were marked red, and the downregulated genes were marked green. The top 100 DEGs were visualized by a heatmap plot.

### WGCNA and Venn diagram

WGCNA was used to identify the key modules of highly correlated genes and explore the relationship between network genes and external sample traits, with the expression data obtained from TCGA and GEO databases. The R package limma (version 3.44.3) was used in checking these expression data, removing duplicate rows, and replenishing missing values. The R package WGCNA (version 1.70.3) was used in analyzing the data. The samples were clustered (cut line as 20,000), and all of the samples were divided into two groups, namely, normal and tumor. Then, the adjacency matrices were transformed into topological overlap matrix (TOM), and the corresponding dissimilarity was calculated (1-TOM). Here, we set the soft-thresholding power as 2 (TCGA-KIRC) and 16 (CEO-ccRCC), cut height as 0.25, and minimal module size as 50. Based on the 1-TOM, the same gene expressions were grouped into a gene co-expression module. Then, important modules were selected, and the intersection with DEGs of TCGA-KIRC and GEO-ccRCC datasets was used. Further analysis, such as GO and KEGG analyses, was conducted on genes that overlapped with those obtained by the four abovementioned datasets.

### Functional annotation and functional enrichment analyses

The R package clusterProfiler (version 3.16.1) (13), org.Hs.eg.db (version 3.11.4), enrichplot (version 1.8.1), and ggplot2 (version 3.3.3) were used in conducting Gene Ontology (GO) enrichment and Kyoto Encyclopedia of Genes and Genomes (KEGG) pathway analyses, with adjusted P< 0.05, a cut-off criterion indicating statistical significance.

### Screening of the prognostic metabolism-related hub gene signature

We intersected the intersection genes with metabolic genes, which were given by the metabolic pathway based on the KEGG online database. Afterward, we obtained the key metabolic genes and adopted univariate Cox regression analysis to screen hub genes associated with prognosis. We regarded P< 0.05 as a significant difference.

### Construction and evaluation of the risk score prognostic model

We obtained the prognostic metabolism-related gene and then performed a Lasso-cox regression analysis to construct a prognostic metabolic-related gene signature. In constructing the risk score prognostic model, we computed the risk score for each patient and divided all the patients into two parts, namely, high-risk patients and low-risk patients, based on the median risk value.


$$\text{Risk score}=(Expr_{gene-1}\times Coef_{gene-1})+(Expr_{gene-2}\times Coef_{gene-2})+\ldots +(Expr_{gene-n}\times Coef_{gene-n})$$

where Expr is the expression of the gene in the signature, and Coef is the Cox coefficient of the gene.

We investigated the time-dependent prognostic significance of the risk score prognostic model using the R package survivalROC (version 1.0.3) and compared it with the predicted effect of age, T, N, M, grade, and stage.

### Verification of the protein level and prognostic values of key genes of the prognostic model

The Human Protein Atlas database (HPA, https://www.proteinatlas.org/), which provides a large amount of transcriptomic and proteomic data in specific human tissues and cells for research, is a valuable database (14). We confirmed the protein level of each key gene between ccRCC and normal tissue using the HPA database, in which immunohistochemistry (IHC) was used to determine protein expression. In addition, we used the UALCAN database (http://ualcan.path.uab.edu/index.html) to confirm the protein level in different stages of ccRCC and normal renal tissues, which provides protein expression analysis option using the data obtained from the Clinical Proteomic Tumor Analysis Consortium (CPTAC) dataset (11). Based on the data obtained from the TCGA database, we used the survival package in R software to explore the prognostic values of key genes and performed Kaplan Meier survival analysis as a box plot graph. Exploring the relationship between disease-free survival (DFS) and the expression of key genes in patients with ccRCC, we used the online tool gene expression profiling interactive analysis (GEPIA, http://gepia.cancer-pku.cn/).

### Assessment of the forecast effect on the risk prognostic model

In validating the association between the risk score and patients’ survival time, we used the pheatmap (version 1.0.12) R package to plot the risk plot and assessed the prognostic value between low- and high-risk patients using the Kaplan-Meier survival curve. We used univariate and multivariate Cox regression analysis to assess the associations between the risk score and various clinicopathological parameters in the TCGA-KIRC Cohort using the Forrest plot. Moreover, exploring the relationship between risk score and tumor grade and stage, we plotted the violin figure using the online tool Sangerbox 3.0 (http://vip.sangerbox.com/). To validate the forecast effect, we randomly sampled 70% of the TCGA-KIRC samples by using the caret (version 6.0.93) R package and formed the validation cohort of this model.

### Prediction of patient’s prognosis and treatment

We used the R package rms (version 6.2.0) to build a predictive nomogram, including clinicopathological characteristics and risk score. Then we used foreign (version 0.8.80) and survival (version 3.3.1) R packages to calculate the concordance index (C-index) and to plot the calibration curves of the predictive nomogram. We also performed a GSEA analysis to identify enriched terms and selected the top 13 significant pathways visualized as the multiple-GSEA plot. pRRopjetic is an R package used for predicting Clinical Chemotherapy (15). We used this R package to predict sensitive drugs for high-risk patients.

### Validation of clinical tissue samples by RT-qPCR experiments

According to the manufacturer’s instructions, we isolated the total RNA of ccRCC tissues and corresponding normal renal tissues using TRIzol (Invitrogen), which was converted into cDNA using PrimeScript™ RT Master Mix (Takara). After cDNA was subjected to reverse transcription PCR using a SYBR-Green master kit (Vazyme, Nanjing, China) on the Applied Biosystems 7500 system, the following cycles were performed: predenaturation at 95°C for 5 min; denaturation at 95°C for 10 s, annealing and extension at 60°C for 34 s; and repeated denaturation, annealing, and extension for 40 cycles. We used β-acting as the housekeeping gene to normalize the relative expression of genes as an endogenous control using the comparative Ct (threshold cycle) method (ΔΔCt). The primers of key genes for the quantitative polymerase chain reaction assay were obtained from Primer Bank, which are shown in **Supplementary Table S1**.

## Results

### Identification of DEG and WGCNA

**Figure 1:**
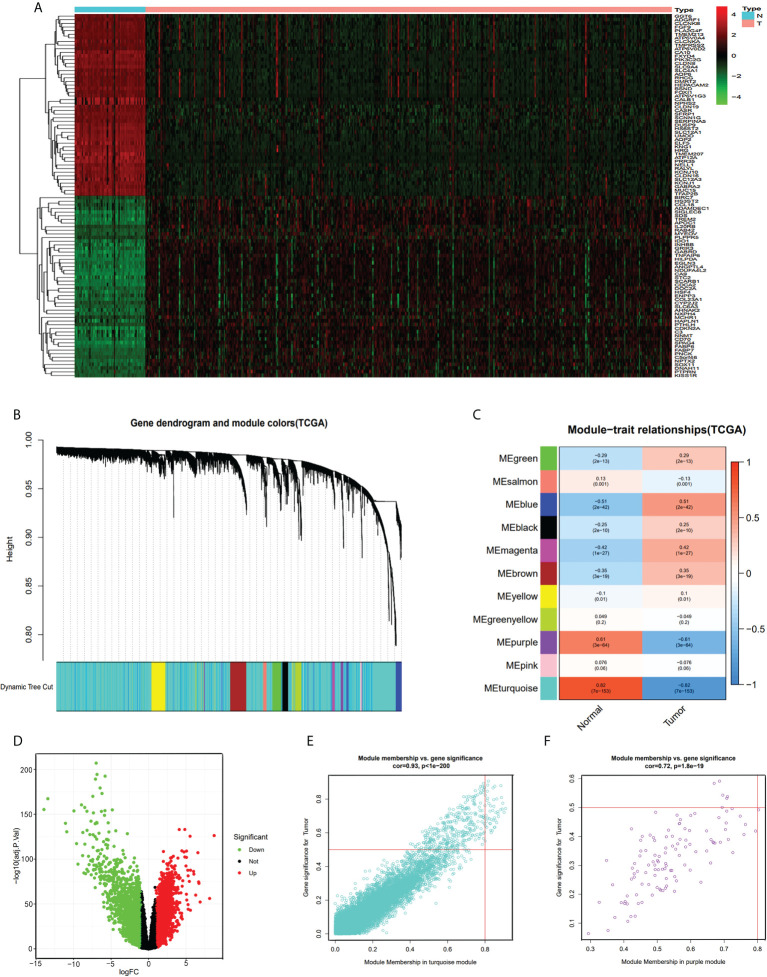
DEG and WGCNA analysis of TCGA-KIRC dataset. **(A)** Heatmap of top 100 DEGs between ccRCC and normal renal tissue from TCGA-KIRC dataset. **(B)** Clustering dendrograms of all the genes of TCGA-KIRC, based on the difference in topological overlap, assigned modules with different colors. **(C)** The eleven co-expression modules and the module trait between ccRCC and normal renal tissue. **(D)** Volcano Plot of 14,684 genes of TCGA-KIRC dataset. Green assigned downregulated genes and rad assigned up. **(E)** Module membership in the turquoise module. **(F)** module membership in the purple module.

We divided the samples of the TCGA-KIRC dataset into two groups, namely, normal group and tumor group, and identified the DEGs with |logFC|>1 and ad. just P<0.05. We screened 3,747 DEGs (1,924 up-regulated and 1,823 down-regulated genes, **Figure 1D**) from 14,684 genes (**Supplementary Table S2**). Then, we selected the top 100 DEGs (50 upregulated and 50 downregulated genes) for visualization by heatmap plot (**Figure 1A**). We used the WGCNA R package to construct weighted gene co-expression modules where each module is assigned with color; a total of 11 modules were included in the TCGA-KIRC (**Figure 1B**). In visualizing the relationship between each module and two clinical traits (normal and tumor), we plotted the heatmap of the module-trait relationship (**Figure 1C**). The genes of each module membership are listed in **Supplementary Table S3**. We found that the turquoise and purple modules were the top two association with clinical traits (MEturquoise module: r=0.82, P=7e-153; MEpurple module: r=0.61, P=3e-64), and the genes of these two modules were downregulated in ccRCC. The relationships between module membership and gene significance are presented in **Figures 1E, F** (turquoise module: cor=0.93, P<1e-200; purple module: cor=0.72, P=1.8e-19).

**Figure 2:**
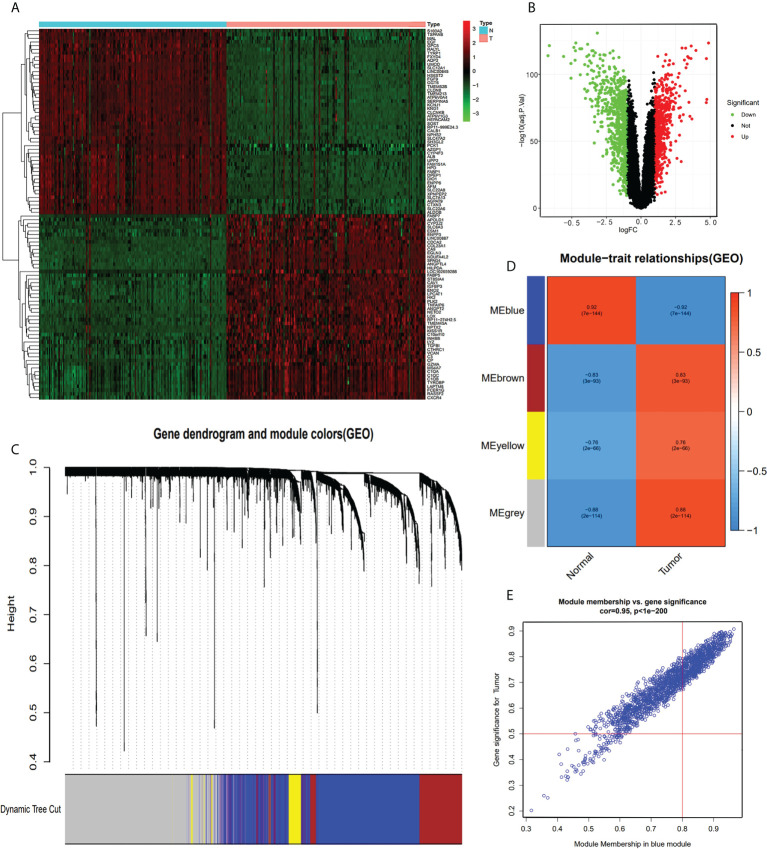
DEG and WGCNA analysis of GEO-ccRCC database. **(A)** Heatmap of top 100 DEGs between ccRCC and normal renal tissue from GEO-ccRCC database. **(B)** Volcano Plot of 21,653 genes of ccRCC in GEO dataset. Green assigned downregulated genes and rad assigned up. **(C)** Clustering dendrograms of all the genes of the GEO-ccRCC database, based on the difference in topological overlap, assigned modules with different colors. **(D)** The four co-expression modules and the module trait between ccRCC and normal renal tissue. **(E)** Module membership in the blue module.

The GEO-ccRCC dataset consisted of four datasets, and all the samples of the database were divided into normal and tumor groups. We identified 1,344 DEGs from 21,653 genes (650 upregulated and 694 downregulated genes, **Figure 2B**) with |logFC|>1 and ad.just P<0.05 (**Supplementary Table S4**). We plotted the heatmap plot for the top 50 upregulated and 50 downregulated genes (**Figure 2A**). Based on the GEO-ccRCC database, we built a total of four weighted gene co-expression modules used in WGCNA analysis (**Figure 2C**), and the heatmap of the module-trait relationship visualized the correlation between each module and clinical traits, namely, normal and tumor (**Figure 2D**). The genes of each module membership are listed in **Supplementary Table S5**. The blue module was the highest relation with clinical traits (r=0.92, P=7e-144), and the relationship between module membership and gene significance for the tumor is visualized and shown in **Figure 2E**.

### Acquisition of overlapping genes and functional enrichment analysis

**Figure 3:**
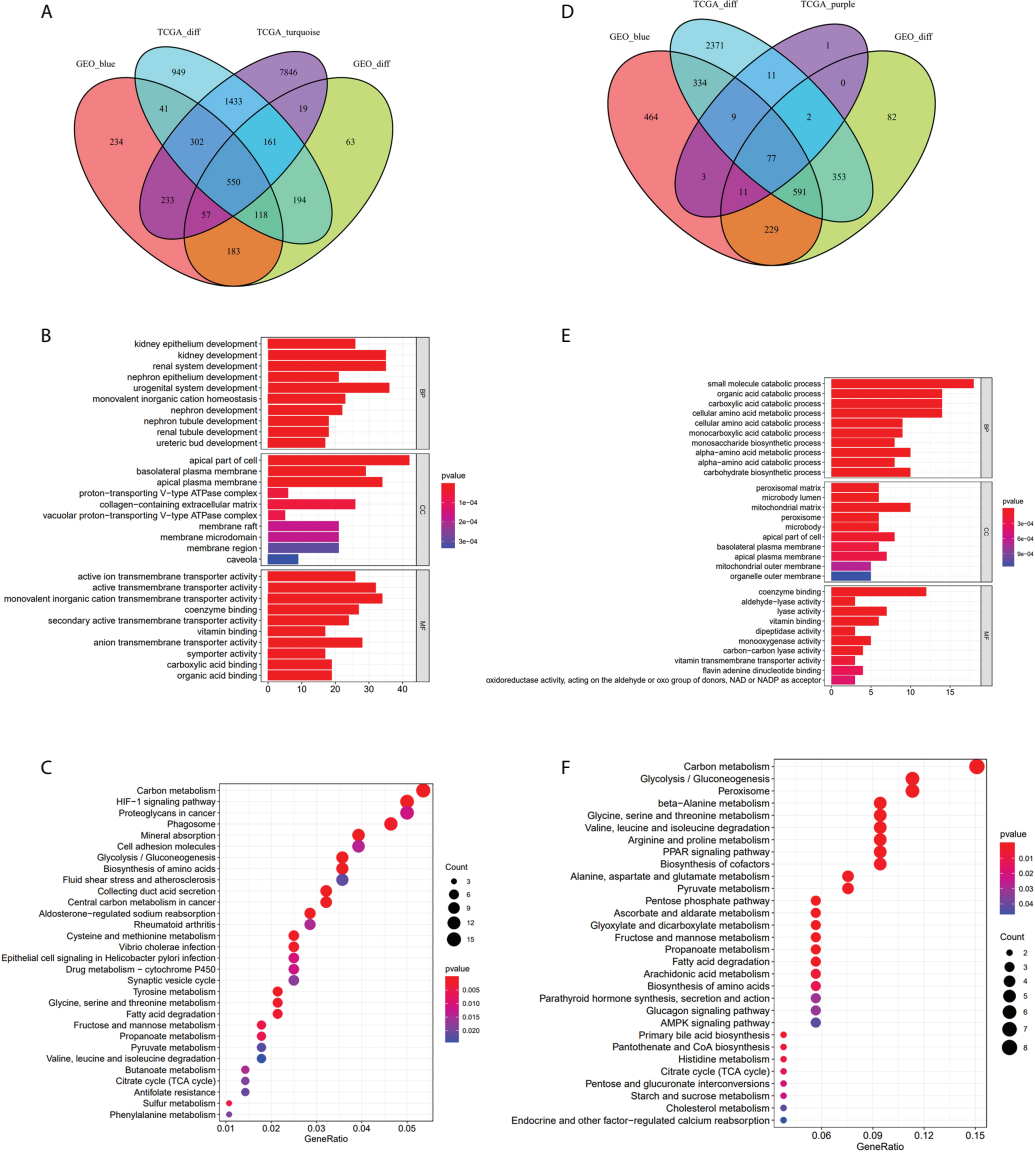
Take the intersection and analysis with GO and KEGG. **(A–C)** The 550 genes given by the intersection of DEGs-TCGA, DEGs-GEO, the turquoise module and the blue module. GO analysis of the 550 genes. KEGG analysis of 550 genes. **(D–F)** The 77 genes obtained from the intersection of DEGs-TCGA, DEGs-GEO, the purple module and the blue module. GO analysis of the 77 genes. KEGG analysis of the 77 genes.

We obtained 3,747 DEGs in the TCGA-KIRC dataset, 1,344 DEGs in the GEO-ccRCC dataset, 10,601 and 114 co-expression genes in the turquoise and purple modules of the TCGA dataset, and 1,717 co-expression genes in the blue module of the GEO dataset. We respectively recorded the intersection of the turquoise and purple modules with DEGs in the TCGA-KIRC dataset, DEGs in the GEO-ccRCC dataset, and the blue module in the GEO dataset. Then, we obtained overlapping 1 (a total of 550 genes, **Figure 3A**) and overlapping 2 (a total of 77 genes, **Figure 3D**) and performed enrichment GO analysis (**Figures 3B, E**) and KEGG analysis (**Figures 3C, F**) on overlapping 1 and overlapping 2. GO enrichment analysis of genes in overlapping 1 showed that the biological process (BP) was primarily enriched in kidney epithelium development, kidney development, and renal system development. In addition, the cellular component (CC) was primarily enriched in the apical part of cell and basolateral plasma membrane, and the molecular function (MF) was primarily gathered in the active ion transmembrane transporter activity and active transmembrane transporter activity (**Supplementary Table S6**). Based on GO analysis of overlapping 2 genes, the BP showed that these genes were primarily enriched in the small-molecule catabolic process, organic acid catabolic process, and carboxylic acid catabolic process. The enrichment of the CC primarily occurred in the peroxisomal matrix and microbody lumen. Moreover, coenzyme binding and aldehyde-lyase activity were more related to these genes in the MF (**Supplementary Table S7**). For KEGG pathway analysis, carbon metabolism and the HIF-1 signaling pathway were associated with overlapping 1 genes (**Supplementary Table S8**). Meanwhile, carbon metabolism, glycolysis/gluconeogenesis, and peroxisome were primarily related to overlapping 2 genes (**Supplementary Table S9**).

### Screening of prognostic metabolism-related hub genes and construction of the risk score prognostic model

**Figure 4:**
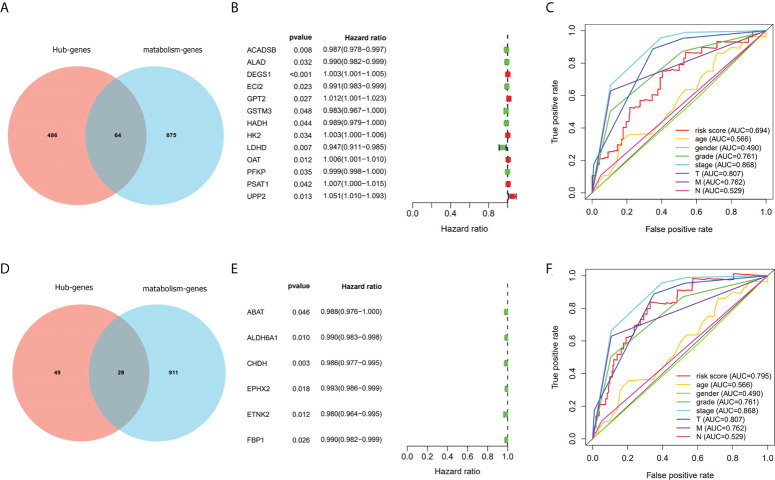
Screen out genes associated with metabolism and prognosis and compare the prognostic model with TNM staging. **(A–C)** Screened prognostic genes gave by turquoise module. **(D–F)** Screened prognostic genes gave by purple module.

As a result of functional enrichment analysis, metabolism and genes in overlapping 1 and 2 have a strong connection. We used the intersection of overlapping 1 and 2 with metabolism-related genes (**Figures 4A, D**) and adopted univariate Cox regression analysis to screen prognostic metabolism-related genes. In overlapping 1, we screened 13 prognostic metabolism-related genes (ACADSB, ALAD, DEGS1, ECI2, GPT2, GSTM3, HADH, HK2, LDHD, OAT, PFKP, PSAT1, and UPP2; **Figure 4B**). Then we constructed the risk score prognostic model 1 (AUC=0.694, **Figure 4C**) with Lasso-cox regression analysis. Meanwhile, we screened six prognostic metabolism-related genes (ABAT, ALDH6A1, CHDH, EPHX2, ETNK2, and FBP1; **Figure 4E**) in overlapping 2 and performed Lasso-cox regression analysis to construct the risk score prognostic model 2 (AUC=0.795, **Figure 4F**). Therefore, we selected the risk score prognostic model 2 and the genes used to construct the model for follow-up analysis.

### Verification of prognostic metabolism-related hub genes

**Figure 5:**
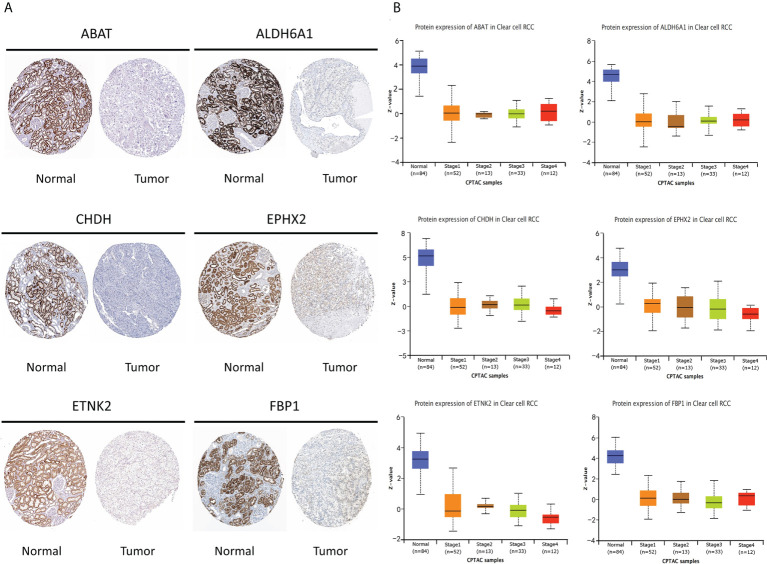
The protein level of the six key genes in kidney normal tissues and ccRCC tissues. **(A)** Immunohistochemical of the six key genes based on The Human Protein Atlas database. **(B)** The protein expression of the six key genes in normal tissues and different stages of ccRCC tissues based on the Clinical Proteomic Tumor Analysis Consortium database.

**Figure 6:**
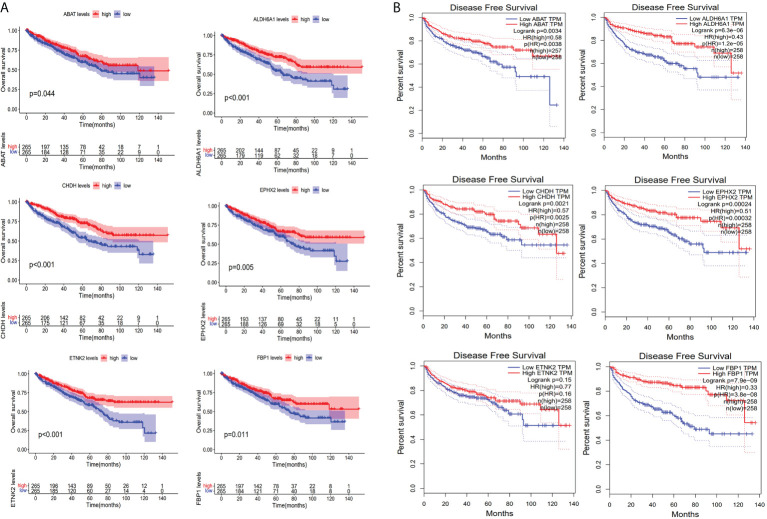
Evaluation of survival. **(A)** Kaplan-Meier survival curves for the six key genes based on the TCGA-KIRC cohort. **(B)** Disease-free survival curves for the six key genes based on Gene expression profiling interactive analysis database.

Based on the immunohistochemical results from the HPA database (**Supplementary Table S10**), the protein level of the six prognostic metabolism-related genes in tumors was generally lower than that in normal tissue (**Figure 5A**). We further verified the protein level of each hub gene in every tumor stage based on the CPTAC dataset (**Figure 5B**). The results of the boxplot showed that the protein level of the six prognostic metabolism-related genes was significantly downregulated in different stages of ccRCC, compared with normal renal tissues. Kaplan-Meier analyses (**Figure 6A**) indicated that the low expression level of each of the six hub genes was significantly associated with poor overall survival (OS) of patients with ccRCC (P<0.05). Moreover, the low expression level of ALDH6A1, CHDH, and ETNK2 was related to worse OS (P<0.001). Meanwhile, based on the GEPIA2 database, we observed that the expression level of ALDH6A1 and FBP1 had a major relationship with worse DFS (P<0.05, **Figure 6B**), in patients with ccRCC.

### Assessment of the predicted effect on the risk score prognostic model

**Figure 7:**
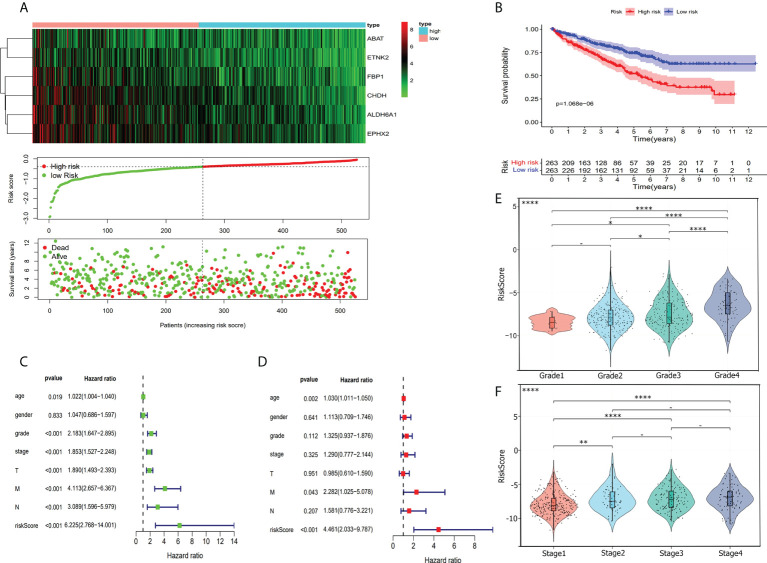
Risk score evaluation. **(A)** To evaluate the prognostic model, we compared the expression of six hub genes in high-risk patients and low-risk patients. Heatmap shows the condition of six hub genes expression. Risk score cove and patients’ survival time plot show the relationship between risks core and patients’ survival time. **(B)** Kaplan-Meier survival curves for high-risk and low-risk patients. **(C)** Forrest plot of the univariate Cox proportional regression analysis in TCGA-KIRC cohort. **(D)** Forrest plot of the multivariate Cox regression analysis in TCGA-KIRC cohort. **(E)** Violin plot shows risk score was closely associated with ccRCC grade. **(F)** Risk score has a relationship with the ccRCC stage. *, *P*< 0.05; **, *P<* 0.01; ****, *P<* 0.0001.

**Figure 8:**
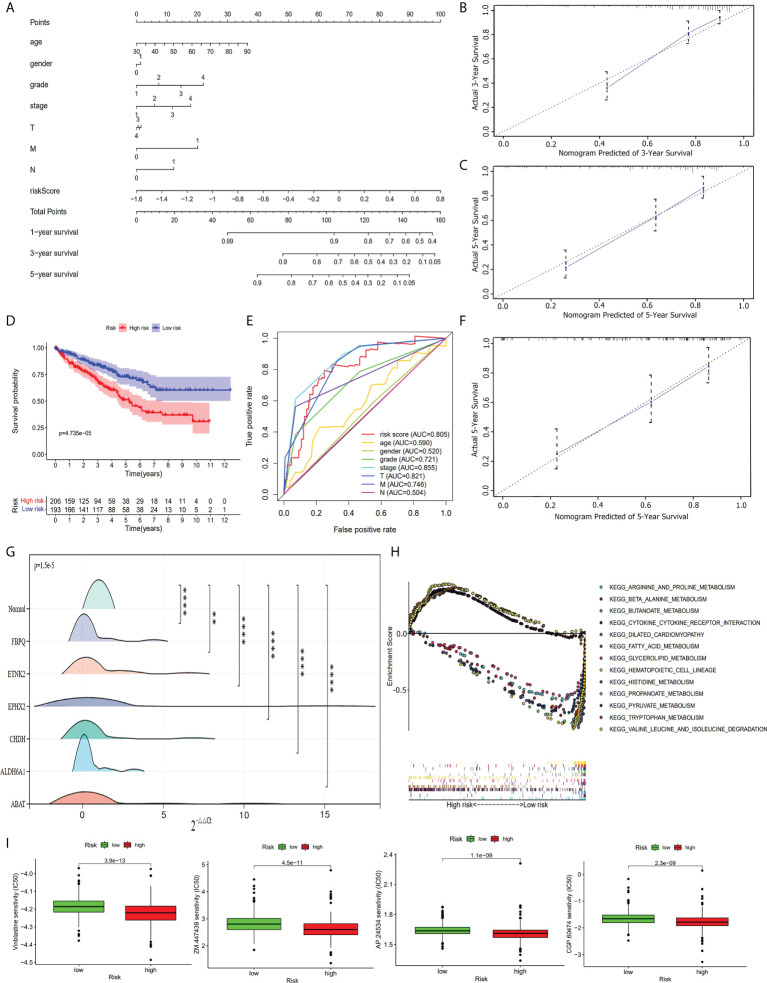
Further analysis of the prognostic model. **(A)** The prognostic nomogram for the prediction of 1- to 5-year overall survival in ccRCC. **(B)** The calibration curves of the nomogram for the prediction of 3-year survival. **(C)** The calibration curves of the nomogram for the prediction of 5-year survival. **(D)** Kaplan-Meier survival curve for high-risk and low-risk patients in the validation cohort. **(E)** ROC curve in the validation cohort. **(F)** The calibration curves of the nomogram for the prediction of 5-year survival in the validation cohort. **(G)** RT-qPCR validation shows the expression of the six hub genes was downregulated in ccRCC tumor tissues. **, *P<* 0.01; ****, *P<* 0.0001. **(H)** Multi-GSEA enrichment analysis shows the top 13 representative KEGG pathways in high-risk and low-risk patients. **(I)** Drug sensitivity analysis shows the most sensitive four drugs in high-risk patients.

In visualizing the correlation between the survival status and risk score in ccRCC patients, we plotted a risk curve (**Figure 7A**), on the basis of the TCGA-KIRC dataset. As shown in **Figure 7A**, patients were divided into high-risk and low-risk groups. The heatmap for the expression of the abovementioned six genes showed that their expression level decreased gradually from the high-risk group to the low-risk group. As the risk score increased, more patients died. Using Kaplan-Meier analysis, we found that the high-risk score was significantly connected related to worse OS (P<0.001, **Figure 7B**). We used univariate Cox regression and multivariate Cox regression analyses to assess the independent role of the risk score prognostic model. Univariate Cox regression analysis (**Figure 7C**) indicated that age, grade, stage, T, M, N, and risk score were correlated with OS, and multivariate Cox regression analysis showed that the risk score could serve as an independent prognostic factor (**Figure 7D**, P<0.001, hazard ratio: 2.033-9.787). **Figures 7E, F** were violin plots for the risk score of different grades and stages of ccRCC tumor. We observed significant differences in risk scores among different ccRCC tumor grades and stages (P<0.001). The prognostic nomogram for the prediction of 1-, 3-, and 5-year survival in ccRCC is shown in **Figure 8A**. By using foreign and survival R packages, we calculated the C-index of the TCGA-KIRC dataset **(**C-index **=** 0.796) and the validation cohort (C-index **=** 0.76). **Figures 8B, C** were calibration curves of the nomogram for predicting patient survival at 3 years and 5 years. We validated the forecast effect using the Kaplan-Meier analysis (P<0.001, **Figure 8D**), ROC curves (risk score AUC = 0.805, **Figure 8E**), and the 5-year survival prediction calibration curve (**Figure 8F**) in the validation cohort.

### GSEA analysis and drug sensitivity test

Based on the TCGA-KIRC dataset, we performed a GSEA analysis to investigate the potential underlying mechanism between the high-risk group and the low-risk group. We enriched 178 upregulated pathways (51 in the high-risk group and 127 in the low-risk group). Of the 127 upregulated pathways in the low-risk group, 43 upregulated pathways were significantly different. The top 13 enriched pathways (three in the high-risk group and ten in the low-risk group) are shown in **Figure 8H**. We used a drug sensitivity test to investigate the sensitive drugs for high-risk patients. The top four major sensitive drugs included Vinblastine sensitivity, ZM.447439, AP.24534, and CGP.60474 (**Figure 8I**).

### RT-qPCR validation of the expression level of the six hub genes

RT-qPCR on 20 paired ccRCC and normal tissue samples (**Supplementary Table S11**) showed that the expression level of the six hub genes in tumor tissues was generally lower than that of normal renal tissues (P<0.05, **Figure 8G**). The six hub genes were significantly downregulated in tumor samples compared with normal samples. This result was consistent with the validated expression level of the six genes that constructed our risk score prognostic model based on an online database.

## Discussion

With the accumulation of cancer research, the link between cancer and various metabolic changes has been revealed. Goldblatt and Cameron obtained transplantable cancer cells from heart fibroblasts through oxygen deficiency experiments (16). Warburg described that the origin of cancer cells from normal tissue cells has two phases: the first phase is an irreversible injury of respiration, and in the second phase the injured cells maintain their structure and energy supply by fermentation energy. Finally, the highly differentiated body cells are converted into undifferentiated cells and grown wildly (17). The fermentation energy that Warburg described in cancer is the earliest mention of metabolic reprogramming, which is not only the beginning but also the propelling of cancers. In addition, he did not fully comprehend the discovery at that time. Approximately 85% of RCC arise from tubular epithelial cells (18). One of the characteristics of RCC is the mutation of genes that are involved in metabolic pathways, including aerobic glycolysis; fatty acid metabolism; and the metabolism of tryptophan, glutamine, and arginine (19). Therefore, RCC is generally regarded as a metabolic disease, and the major risk factors include aerobic glycolysis and the mutation of metabolic genes (20, 21). In this study, we characterized ccRCC to explore the prognostic prediction model and treatment of ccRCC. We performed bioinformatics analysis to screen out prognostic metabolism-related key genes and construct the risk score prognostic model using these key genes.

To avoid errors caused by insufficient sample size, the ccRCC samples in this study were obtained from TCGA and GEO databases, a total of 965 samples. we used DEG analysis and WGCNA to screen the clinical traits related to DEGs. Based on the correlation coefficient of genes, WGCNA, a network analysis method, can identify biologically relevant modules and key genes (5), which are correlated with clinical traits. Such modules and key genes may serve as biomarkers for detection or treatment (22). Therefore, WGCNA has unique advantages in exploring the relationship of clinical traits related to modules. We found that the coexpression modules closely related to clinical traits were downregulated in ccRCC, and the functional enrichment analysis showed that the genes in those modules were related to metabolism. This result is in line with our expected results. Finally, the genes of the risk score prognostic model that we constructed included ABAT, ALDH6A1, CHDH, EPHX2, ETNK2, and FBP1, which were downregulated in ccRCC. Multivariate Cox regression analysis showed that the risk score of the prognostic model could serve as an independent prognostic factor. To compare the predicted effect of the risk score prognostic model with that of age, T, N, M, grade, and stage, we plotted ROC curves by the TCGA-KIRC cohort and the validation cohort. The results of ROC curves showed that the predicted effect of the risk score prognostic model was similar to that of clinical grade and stage. The prognostic nomogram combined abundant factors, including age, T, N, M, grade, stage, and risk score. By using the nomogram, we can make a more accurate prediction of 1-, 3-, and 5-year survival of ccRCC patients.

The six key genes are involved in various metabolic reactions, including amino acid metabolism, choline metabolism, and glucose metabolism. Most of the genes are involved in cancers. The 4-aminobutyrate aminotransferase (ABAT) encodes γ-aminobutyric acid (GABA) transaminase, which is a key enzyme for catabolism GABA, a major inhibitory neurotransmitter, within the mitochondrial matrix. ABAT plays an important role in neurometabolic disorders (23). The deficiency of ABAT mediates the destruction of the GABAergic system, and patients present corresponding clinical manifestations of elevated GABA levels (24). For breast cancer, the loss of ABAT expression could promote the potency of tumorigenesis and metastasis (25), which could be a predictive biomarker for endocrine therapy resistance (26). The results of our study show that the expression of ABAT was downregulated in ccRCC and related with poor DFS of patients. GSEA analysis shows that the beta alanine metabolism pathway associated with ABAT was upregulated in the low-risk group. A previous study indicated that ABAT and aldehyde dehydrogenase 6 family member A1 (ALDH6A1) worked as a tumor suppressor (27) in ccRCC, thereby suppressing tumorigenic capability. In this study, the downregulation of the expression of ALDH6A1, an amino acid metabolism-related gene, was significantly linked with worse OS and DFS in patients. Meanwhile, ALDH6A1 was identified as a potential molecular signature for hepatocellular carcinoma (28), prostate cancer (29), and muscle insulin resistance in type 2 diabetes mellitus (30). Therefore, ALDH6A1 may be a potential key target for regulating ccRCC metabolism. Based on the results of the GSEA analysis, ABAT and ALDH6A1 function in the valine leucine and isoleucine degradation pathways, which are upregulated in the low-risk group. In addition, ABAT plays a role in the butanoate metabolism and alanine aspartate and glutamate metabolism pathways. The physiological role of Human choline dehydrogenase (CHDH) is to regulate the concentration of choline and glycine betaine, and CHDH is primarily located in the liver and kidney (31). Moreover, CHDH plays a pivotal role in mitophagy (32). Based on our results, genes of glycine serine and threonine metabolism pathway, including CHDH, and the downregulation of CDHD in ccRCC, were related to worse OS and DFS in patients. Soluble epoxide hydrolase (EPHX2, sEH) serves as a principal enzyme for the metabolism of epoxyeicosatrienoic acids (33), and it is related to cell apoptosis (34). For metabolic diseases, EPHX2 may be a potential therapeutic target (35). In prostate cancer and hepatocellular carcinoma, EPHX2 was downregulated, which was significantly correlated with the progression of tumors (36, 37). Based on the enrichment results of GSEA, EPHX2 is intimate with the peroxisome pathway. Furthermore, the mechanism of CHDH and EPHX2 in ccRCC was deficient. Ethanolamine kinase 2 (ETNK2) was also reported in tumors. The upregulation of ETNK2 enhances hepatic metastasis such as gastric cancer (38). However, ETNK2 was downregulated in our research for ccRCC with a poor OS of patients. Fructose-1, 6-bisphosphatase 1 (FBP1), a rate-limiting enzyme for gluconeogenesis (39), plays a critical role in tumor initiation and progression of ccRCC. FBP1 has two major mechanisms that inhibit ccRCC progression: first, FBP1 can inhibit a potential Warburg effect; second, FBP1 can interact with the HIF inhibitory domain and inhibit nuclear HIF function (40). Targeting FBP1 has been an emerging therapeutical target for cancers (39). Thus, this study aimed to explore more therapeutical targets for ccRCC *via* screening the prognostic metabolism-related genes.

Using the six prognostic metabolism-related genes, we constructed a risk score prognostic model and divided patients with ccRCC into high- and low-risk groups based on the risk score of each patient. We found that the patients in the high-risk group have poor OS, and lass survival time. Based on the violin plots, we discovered that the risk scores on each grade and stage of ccRCC were significantly different, and the risk scores displayed a significantly positive correlation with the degree of malignancy of ccRCC. The results of multivariate Cox regression analysis showed that the risk score of the prognostic model could serve as an independent prognostic factor. Based on the prediction of clinical chemotherapeutic response analysis, we screened four drugs, namely, Vinblastine, ZM.447439, AP.24534, and CGP.60474, which may be more sensitive for patients in the high-risk group. Vinblastine is a dimeric alkaloid isolated from the Madagascar periwinkle plant, which exhibits significant cytotoxic activity, and it is used as an antineoplastic agent in antitumor therapy (41). For our analysis, Vinblastine is the principal sensitive drug for high-risk group patients. Mitotic Aurora kinases are essential for accurate chromosomal segregation during cell division. As an Aurora-selective ATP-competitive inhibitor, ZM.447439 can interfere with the spindle integrity checkpoint and chromosomal segregation (42). In addition, Vinblastine and ZM.447439 function by interfering with cancer cell proliferation. AP.24534 and CGP.60474 are kinase inhibitors. Ponatinib, AP.24534, is a third-generation tyrosine kinase receptor inhibitor (43), and CGP.60474 is an inhibitor of cyclin-dependent kinase (44). Tyrosine kinase inhibitors are novel therapies for ccRCC treatment (45, 46), including sunitinib, sorafenib, pazopanib, axitinib, and tivozanib (47–50). Tyrosine kinases are signaling molecules, and tyrosine kinase inhibitors have become a successful class of drugs in the treatment of ccRCC. Thus, we might consider that these four identified sensitive drugs could be potential treatments for ccRCC, and we believe that novel drugs worked by regulating the pathway of cellular metabolism will appear increasingly in the near future.

This research also has some limitations. First, this is a retrospective study, we need more clinical samples to improve our findings and the predicted effect of the risk score prognostic model. Second, the molecular mechanisms of the six key genes need to be further elucidated *in vivo* and *in vitro* experiments for ccRCC clinical applications.

## Conclusions

In conclusion, based on the metabolic reprogramming characteristics in ccRCC and combined with WGCNA analysis, we identified six metabolism-related genes, which could be potential treatment targets for ccRCC. Furthermore, we constructed a risk score prognostic model, the risk score of which constitutes an effective independent prognostic factor. By including the risk score, the nomogram can help us make a more accurate prediction of patient survival. The improvement of the prognostic model may improve the outcome prediction for ccRCC patients in the future.

## Data availability statement

The datasets presented in this study can be found in online repositories. The names of the repository/repositories and accession number(s) can be found in the article/**Supplementary Material**.

## Author contributions

Conception and design: JL, CL, QZ, and LD. Administrative support: JL and CL. Provision of study materials or patients: CL, QZ, and LD. Collection and assembly of data: QDZ and CN. Data analysis and interpretation: CL, QZ, TZ, and LD. Manuscript writing: All authors. All authors contributed to the article and approved the submitted version.

## Funding

This study was supported by the National Natural Science Foundation of China (Grant no. 82002718); and the Jiangsu Natural Science Foundation (Grant no. BK20191077).

## Acknowledgments

The authors highly appreciate all the members of Biomathematics for improvement of this manuscript.

## Conflict of interest

The authors declare that the research was conducted in the absence of any commercial or financial relationships that could be construed as a potential conflict of interest.

## Publisher’s note

All claims expressed in this article are solely those of the authors and do not necessarily represent those of their affiliated organizations, or those of the publisher, the editors and the reviewers. Any product that may be evaluated in this article, or claim that may be made by its manufacturer, is not guaranteed or endorsed by the publisher.

## References

[1] Sung H Ferlay J Siegel RL Laversanne M Soerjomataram I Jemal A . Global cancer statistics 2020: Globocan estimates of incidence and mortality worldwide for 36 cancers in 185 countries. CA Cancer J Clin (2021) 71(3):209–49. doi: 10.3322/caac.21660 33538338

[2] Siegel RL Miller KD Fuchs HE Jemal A . Cancer statistics, 2021. CA Cancer J Clin (2021) 71(1):7–33. doi: 10.3322/caac.21654 33433946

[3] Motzer RJ Ravaud A Patard JJ Pandha HS George DJ Patel A . Adjuvant sunitinib for high-risk renal cell carcinoma after nephrectomy: Subgroup analyses and updated overall survival results. Eur Urol (2018) 73(1):62–8. doi: 10.1016/j.eururo.2017.09.008 PMC6684251 28967554

[4] Ayyildiz D Piazza S . Introduction to bioinformatics. Methods Mol Biol (2019) 1986:1–15. doi: 10.1007/978-1-4939-9442-7_1 31115882

[5] Langfelder P Horvath S . Wgcna: An r package for weighted correlation network analysis. BMC Bioinf (2008) 9:559. doi: 10.1186/1471-2105-9-559 PMC2631488 19114008

[6] Weiss RH . Metabolomics and metabolic reprogramming in kidney cancer. Semin Nephrol (2018) 38(2):175–82. doi: 10.1016/j.semnephrol.2018.01.006 PMC6009840 29602399

[7] Wettersten HI Aboud OA Lara PN Jr. Weiss RH . Metabolic reprogramming in clear cell renal cell carcinoma. Nat Rev Nephrol (2017) 13(7):410–9. doi: 10.1038/nrneph.2017.59 28480903

[8] Sanchez DJ Simon MC . Genetic and metabolic hallmarks of clear cell renal cell carcinoma. Biochim Biophys Acta Rev Cancer (2018) 1870(1):23–31. doi: 10.1016/j.bbcan.2018.06.003 29959988 PMC6561085

[9] Qiu B Ackerman D Sanchez DJ Li B Ochocki JD Grazioli A . Hif2alpha-dependent lipid storage promotes endoplasmic reticulum homeostasis in clear-cell renal cell carcinoma. Cancer Discovery (2015) 5(6):652–67. doi: 10.1158/2159-8290.CD-14-1507 PMC4456212 25829424

[10] Gentleman RC Carey VJ Bates DM Bolstad B Dettling M Dudoit S . Bioconductor: Open software development for computational biology and bioinformatics. Genome Biol (2004) 5(10):R80. doi: 10.1186/gb-2004-5-10-r80 15461798 PMC545600

[11] Jones L Goldstein DR Hughes G Hu Y Law CW Shi W . Assessment of the relationship between pre-chip and post-chip quality measures for affymetrix genechip expression data. BMC Bioinf (2006) 7:211. doi: 10.1186/1471-2105-7-211 PMC1524996 16623940

[12] Ritchie ME Phipson B Wu D Hu Y Law CW Shi W . Limma powers differential expression analyses for rna-sequencing and microarray studies. Nucleic Acids Res (2015) 43(7):e47. doi: 10.1093/nar/gkv007 25605792 PMC4402510

[13] Yu G Wang LG Han Y He QY . Clusterprofiler: An r package for comparing biological themes among gene clusters. OMICS (2012) 16(5):284–7. doi: 10.1089/omi.2011.0118 PMC3339379 22455463

[14] Thul PJ Lindskog C . The human protein atlas: A spatial map of the human proteome. Protein Sci (2018) 27(1):233–44. doi: 10.1002/pro.3307 PMC5734309 28940711

[15] Geeleher P Cox N Huang RS . Prrophetic: An r package for prediction of clinical chemotherapeutic response from tumor gene expression levels. PloS One (2014) 9(9):e107468. doi: 10.1371/journal.pone.0107468 25229481 PMC4167990

[16] GOLDBLATT H CAMERON G . Induced malignancy in cells from rat myocardium subjected to intermittent anaerobiosis during long propagation *in vitro* . J Exp Med (1953) 97(4):525–52. doi: 10.1084/jem.97.4.525 PMC2136288 13052818

[17] WARBURG O . On the origin of cancer cells. Science (1956) 123(3191):309–14. doi: 10.1126/science.123.3191.309 13298683

[18] Cohen HT McGovern FJ . Renal-cell carcinoma. New Engl J Med (2005) 353(23):2477–90. doi: 10.1056/NEJMra043172 16339096

[19] Lucarelli G Loizzo D Franzin R Battaglia S Ferro M Cantiello F . Metabolomic insights into pathophysiological mechanisms and biomarker discovery in clear cell renal cell carcinoma. Expert Rev Mol Diagn (2019) 19(5):397–407. doi: 10.1080/14737159.2019.1607729 30983433

[20] Nickerson ML Jaeger E Shi Y Durocher JA Mahurkar S Zaridze D . Improved identification of von hippel-lindau gene alterations in clear cell renal tumors. Clin Cancer Res (2008) 14(15):4726–34. doi: 10.1158/1078-0432.CCR-07-4921 PMC2629664 18676741

[21] Harlander S Schonenberger D Toussaint NC Prummer M Catalano A Brandt L . Combined mutation in vhl, trp53 and rb1 causes clear cell renal cell carcinoma in mice. Nat Med (2017) 23(7):869–77. doi: 10.1038/nm.4343 PMC5509015 28553932

[22] Long J Huang S Bai Y Mao J Wang A Lin Y . Transcriptional landscape of cholangiocarcinoma revealed by weighted gene coexpression network analysis. Brief Bioinform (2021) 22(4):bbaa224. doi: 10.1093/bib/bbaa224 33051665

[23] Besse A Wu P Bruni F Donti T Graham BH Craigen WJ . The gaba transaminase, abat, is essential for mitochondrial nucleoside metabolism. Cell Metab (2015) 21(3):417–27. doi: 10.1016/j.cmet.2015.02.008 PMC4757431 25738457

[24] Jaeken J Casaer P de Cock P Corbeel L Eeckels R Eggermont E . Gamma-aminobutyric acid-transaminase deficiency: A newly recognized inborn error of neurotransmitter metabolism. Neuropediatrics (1984) 15(3):165–9. doi: 10.1055/s-2008-1052362 6148708

[25] Chen X Cao Q Liao R Wu X Xun S Huang J . Loss of abat-mediated gabaergic system promotes basal-like breast cancer progression by activating ca(2+)-nfat1 axis. Theranostics (2019) 9(1):34–47. doi: 10.7150/thno.29407 30662552 PMC6332792

[26] Jansen MPHM Sas L Sieuwerts AM Van Cauwenberghe C Ramirez-Ardila D Look M . Decreased expression of abat and stc2 hallmarks er-positive inflammatory breast cancer and endocrine therapy resistance in advanced disease. Mol Oncol (2015) 9(6):1218–33. doi: 10.1016/j.molonc.2015.02.006 PMC5528763 25771305

[27] Lu J Chen Z Zhao H Dong H Zhu L Zhang Y . Abat and aldh6a1, regulated by transcription factor hnf4a, suppress tumorigenic capability in clear cell renal cell carcinoma. J Trans Med (2020) 18(1):101. doi: 10.1186/s12967-020-02268-1 PMC7038561 32093682

[28] Shin H Cha HJ Lee MJ Na K Park D Kim CY . Identification of aldh6a1 as a potential molecular signature in hepatocellular carcinoma *via* quantitative profiling of the mitochondrial proteome. J Proteome Res (2020) 19(4):1684–95. doi: 10.1021/acs.jproteome.9b00846 31985234

[29] Cho SY Kang S Kim DS Na HJ Kim YJ Choi YD . Hsp27, aldh6a1 and prohibitin act as a trio-biomarker to predict survival in late metastatic prostate cancer. Anticancer Res (2018) 38(11):6551–60. doi: 10.21873/anticanres.13021 30396985

[30] Liu S Cai X Wang T Xu J Cheng W Wang X . Downregulation of aldh6a1 is a new marker of muscle insulin resistance in type 2 diabetes mellitus. Int J Gen Med (2022) 15:2137–47. doi: 10.2147/IJGM.S343727 PMC8887615 35241929

[31] Salvi F . Gadda G human choline dehydrogenase: Medical promises and biochemical challenges. Arch Biochem biophysics (2013) 537(2):243–52. doi: 10.1016/j.abb.2013.07.018 PMC7094428 23906661

[32] Park S Choi SG Yoo SM Son JH Jung YK . Choline dehydrogenase interacts with sqstm1/p62 to recruit lc3 and stimulate mitophagy. Autophagy (2014) 10(11):1906–20. doi: 10.4161/auto.32177 PMC4502719 25483962

[33] Wang YX Ulu A Zhang LN Hammock B . Soluble epoxide hydrolase in atherosclerosis. Curr Atheroscler Rep (2010) 12(3):174–83. doi: 10.1007/s11883-010-0108-5 PMC2857794 20425256

[34] Li X Wu X . Soluble epoxide hydrolase (ephx2) silencing attenuates the hydrogen peroxide-induced oxidative damage in iec-6 cells. Arch Med Sci (2021) 17(4):1075–86. doi: 10.5114/aoms.2019.87137 PMC8314398 34336035

[35] He J Wang C Zhu Y Ai D . Soluble epoxide hydrolase: A potential target for metabolic diseases. J Diabetes (2016) 8(3):305–13. doi: 10.1111/1753-0407.12358 26621325

[36] Liu MS Zhao H Xu CX Xie PB Wang W Yang YY . Clinical significance of ephx2 deregulation in prostate cancer. Asian J andrology (2021) 23(1):109–15. doi: 10.4103/aja.aja_34_20 PMC7831821 32687069

[37] Zhan K Bai Y Liao S Chen H Kuang L Luo Q . Identification and validation of ephx2 as a prognostic biomarker in hepatocellular carcinoma. Mol Med Rep (2021) 24(3):650. doi: 10.3892/mmr.2021.12289 34278494 PMC8299194

[38] Miwa T Kanda M Shimizu D Umeda S Sawaki K Tanaka H . Hepatic metastasis of gastric cancer is associated with enhanced expression of ethanolamine kinase 2 *via* the p53-bcl-2 intrinsic apoptosis pathway. Br J Cancer (2021) 124(8):1449–60. doi: 10.1038/s41416-021-01271-7 PMC8039033 33531692

[39] Liu GM . Zhang YM targeting fbpase is an emerging novel approach for cancer therapy. Cancer Cell Int (2018) 18:36. doi: 10.1186/s12935-018-0533-z 29556139 PMC5845355

[40] Li B Qiu B Lee DS Walton ZE Ochocki JD Mathew LK . Fructose-1,6-bisphosphatase opposes renal carcinoma progression. Nature (2014) 513(7517):251–5. doi: 10.1038/nature13557 PMC4162811 25043030

[41] Keglevich P Hazai L Kalaus G Szantay C . Modifications on the basic skeletons of vinblastine and vincristine. Molecules (2012) 17(5):5893–914. doi: 10.3390/molecules17055893 PMC6268133 22609781

[42] Long ZJ Xu J Yan M Zhang JG Guan Z Xu DZ . Zm 447439 inhibition of aurora kinase induces hep2 cancer cell apoptosis in three-dimensional culture. Cell Cycle (2008) 7(10):1473–9. doi: 10.4161/cc.7.10.5949 18418083

[43] Massaro F Molica M Breccia M . Ponatinib: A review of efficacy and safety. Curr Cancer Drug Targets (2018) 18(9):847–56. doi: 10.2174/1568009617666171002142659 28969556

[44] Han HW Hahn S Jeong HY Jee JH Nam MO Kim HK . Lincs l1000 dataset-based repositioning of cgp-60474 as a highly potent anti-endotoxemic agent. Sci Rep (2018) 8(1):14969. doi: 10.1038/s41598-018-33039-0 30297806 PMC6175892

[45] Gupta K Miller JD Li JZ Russell MW Charbonneau C . Epidemiologic and socioeconomic burden of metastatic renal cell carcinoma (mrcc): A literature review. Cancer Treat Rev (2008) 34(3):193–205. doi: 10.1016/j.ctrv.2007.12.001 18313224

[46] Bielecka ZF Czarnecka AM Solarek W Kornakiewicz A Szczylik C . Mechanisms of acquired resistance to tyrosine kinase inhibitors in clear - cell renal cell carcinoma (ccRCC). Curr Signal transduction Ther (2014) 8(3):218–28. doi: 10.2174/1574362409666140206223014 PMC4141325 25152704

[47] Escudier B Eisen T Stadler WM Szczylik C Oudard S Siebels M . Sorafenib in advanced clear-cell renal-cell carcinoma. New Engl J Med (2007) 356(2):125–34. doi: 10.1056/NEJMoa060655 17215530

[48] Motzer RJ Hutson TE Tomczak P Michaelson MD Bukowski RM Rixe O . Sunitinib versus interferon alfa in metastatic renal-cell carcinoma. New Engl J Med (2007) 356(2):115–24. doi: 10.1056/NEJMoa065044 17215529

[49] Sternberg CN Davis ID Mardiak J Szczylik C Lee E Wagstaff J . Pazopanib in locally advanced or metastatic renal cell carcinoma: Results of a randomized phase iii trial. J Clin Oncol Off J Am Soc Clin Oncol (2010) 28(6):1061–8. doi: 10.1200/JCO.2009.23.9764 20100962

[50] Motzer RJ Nosov D Eisen T Bondarenko I Lesovoy V Lipatov O . Tivozanib versus sorafenib as initial targeted therapy for patients with metastatic renal cell carcinoma: Results from a phase iii trial. J Clin Oncol Off J Am Soc Clin Oncol (2013) 31(30):3791–9. doi: 10.1200/JCO.2012.47.4940 PMC5569677 24019545

