# Supplementary figures and images for: A metabolic reprogramming-related prognostic risk model for clear cell renal cell carcinoma: From construction to preliminary application
Source: Front Oncol. 2022 Sep 13;12:982426. doi: 10.3389/fonc.2022.982426 (PMC9513462; doi:10.3389/fonc.2022.982426)

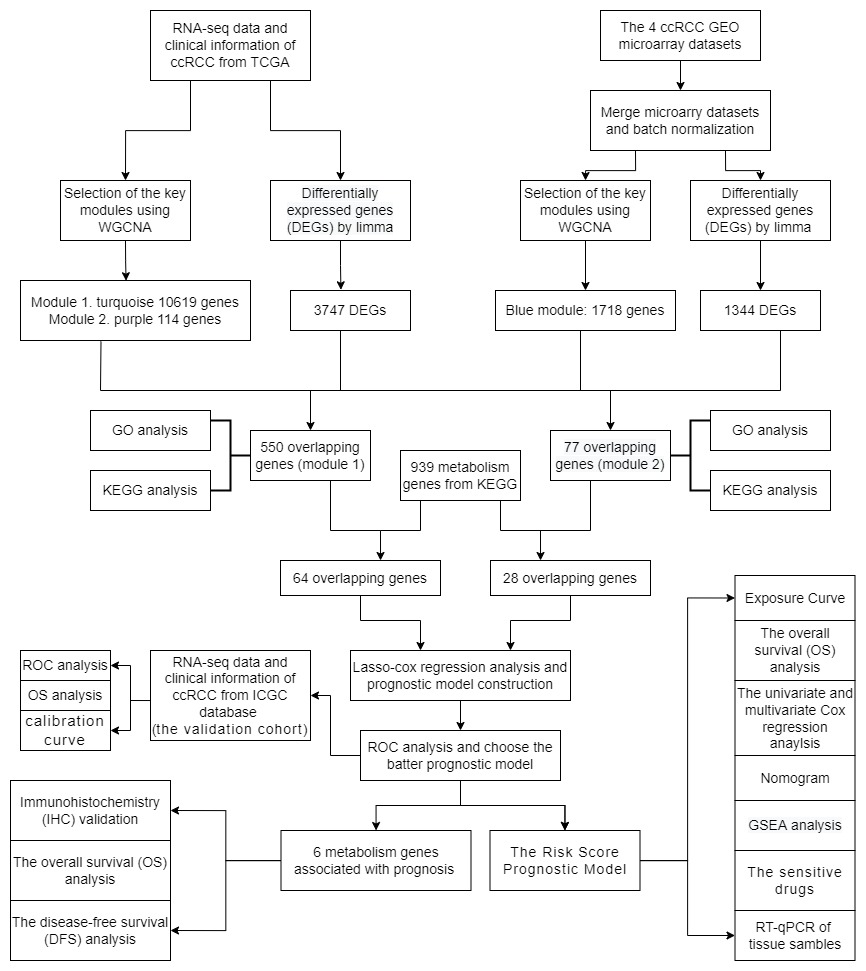

Supplement: Supplementary file 2 [file Image_1.jpeg]
